# Synthesis of Carbon Nanotube (CNT) Composite Membranes

**DOI:** 10.3390/membranes1010037

**Published:** 2010-12-27

**Authors:** Tariq Altalhi, Milena Ginic-Markovic, Ninghui Han, Stephen Clarke, Dusan Losic

**Affiliations:** 1 School of Physical and Chemical Science, Flinders University, Adelaide, SA 5000, Australia; E-Mails: alta0013@flinders.edu.au (T.A.); stephen.clarke@flinders.edu.au (S.K.); 2 Ian Wark Research Institute, University of South Australia, Mawson Lakes, Adelaide, SA 5095, Australia; E-Mails: hnhzzh@yahoo.com.cn (N.H); dusan.losic@unisa.edu.au (D.L.)

**Keywords:** carbon nanotubes, nanoporous alumina membranes, electrochemical anodization, catalyst free carbon precursor, transport properties

## Abstract

Carbon nanotubes are attractive approach for designing of new membranes for advanced molecular separation because of their unique transport properties and ability to mimic biological protein channels. In this work the synthetic approach for fabrication of carbon nanotubes (CNTs) composite membranes is presented. The method is based on growth of multi walled carbon nanotubes (MWCNT) using chemical vapour deposition (CVD) on the template of nanoporous alumina (PA) membranes. The influence of experimental conditions including carbon precursor, temperature, deposition time, and PA template on CNT growth process and quality of fabricated membranes was investigated. The synthesis of CNT/PA composites with controllable nanotube dimensions such as diameters (30–150 nm), and thickness (5–100 μm), was demonstrated. The chemical composition and morphological characteristics of fabricated CNT/PA composite membranes were investigated by various characterisation techniques including scanning electron microscopy (SEM), energy-dispersive x-ray spectroscopy (EDXS), high resolution transmission electron microscopy (HRTEM) and x-ray diffraction (XRD). Transport properties of prepared membranes were explored by diffusion of dye (Rose Bengal) used as model of hydrophilic transport molecule.

## 1. Introduction

Porous membranes with nanoscale pore diameters have attracted considerable interest in the last two decades for applications in various fields such as molecular separation, adsorption, catalysis, energy storage, biosensing, cell culture, template synthesis and drug delivery [1-5]. The most critical issue in designing of nanoporous membranes is to optimise their chemical selectivity and high transport properties. Numerous approaches have been investigated to achieve these goals, exploring membranes made from different nanomaterials including zeolites, silica, inorganic oxides, carbon, metals, metallo-organic composites, and polymers [6-7]. Demonstration of molecular separations has been achieved by nanometer-scale control of porous alumina (PA) membranes pore dimensions. The control of the pores dimensions has been accomplished using the plating of nanoporous polycarbonate ion track-etch and ordered porous alumina (PA) membranes with initial pore dimensions of 20 to 50 nm [2,8-9].

Carbon nanotubes (CNTs) have recently attracted great attention for development of new membranes with advanced transport and selectivity features because of their unique properties such as temperature stability, mechanical strengths, chemical inertness, conductivity and most importantly outstanding water transport properties [10-11]. In principle, the inner cores of carbon nanotubes (CNTs) can enable fine control of pore dimension at the nanometer scale. The transport rates of gases and water molecules through CNTs has been found in one to two orders of magnitude larger than in the zeolitic pores or in any microporous material. This phenomenon has been explained to CNTs' extremely friction-less graphitic interfaces and smooth, defect-free walls [11]. Recently, the fabrication of CNT membranes with arrays of single CNTs has been successfully demonstrated by embedding CNT by polymer film. However, there are many problems with this fabrication approach [11-13]. To align large numbers of CNTs nanotubes and prepare a robust membrane structure with well-controlled nanometer scale dimension is a substantial challenge. The fabrication of these membranes assembly is in particular time consuming, expensive and difficult to scale-up; to make CNT membranes using inexpensive and feasible for practical application.

To address these problems and make CNT membranes fabrication process more feasible, less-expensive and mechanically stronger a new approach for growth of CNT inside of inorganic scaffold such as nanoporous alumina membranes (PA) is recently demonstrated. PA template-confined growth of CNTs is described to be an efficient approach to the production of aligned arrays of CNTs that can be used for many applications not only for molecular separations [14-16]. The approach is based on carbonization of polymers or pyrolysis of gaseous hydrocarbons to produce CNTs inside of PA pores [16-18].

**Figure 1:**
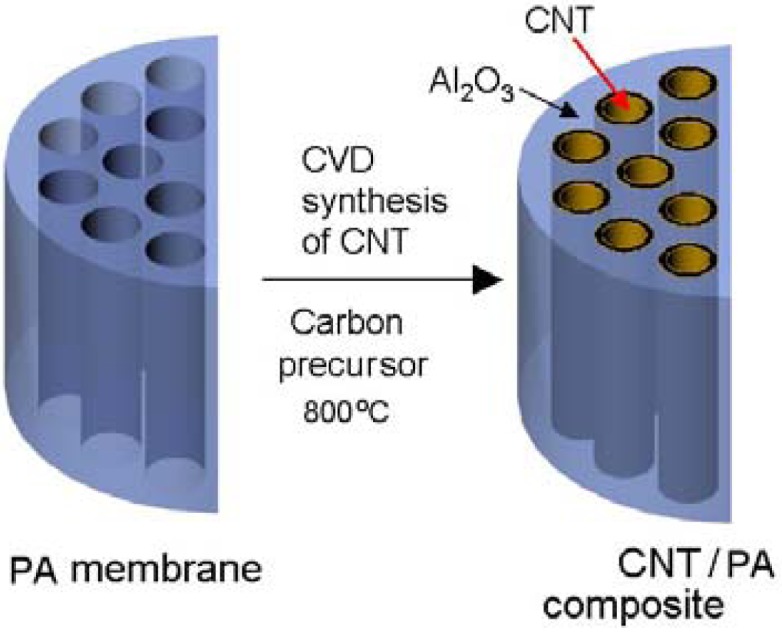
Schematic of fabrication CNT/PA membrane composites by CVD growth of carbon nanotubes inside of porous alumina oxide pores.

In this work we present the method for fabrication of multi waled CNT (MWCNT) using chemical vapour deposition (CVD) on the template of nanoporous alumina membranes. The Schematic of the fabrication method is presented in Figure 1 showing the model of PA pore structure before and the growth of CNT structure inside of pores. The influence of experimental conditions including different carbon precursor (with catalyst and catalyst free), temperature, and deposition time on CNT growth process was investigated in order to optimise fabrication protocol and quality of fabricated membranes. The structural and chemical composition of fabricated CNT/PA composite membranes were investigated by various characterisation techniques including scanning electron microscopy (SEM), energy-dispersive x-ray spectroscopy (EDXS), high resolution transmission electron microscopy (HRTEM), x-ray diffraction (XRD), and x-ray photoelectron spectroscopy (XPS). Transport properties of prepared membranes were explored by diffusion experiment using dye (Rose Bengal) as model molecule.

## 2. Experimental Section

### 2.1. Materials

A high purity (99.997%) aluminium foil supplied from Alfa Aesar (USA) was used as the substrate material. The chemicals used in this work including oxalic acid, phosphoric acid ferrocene, cupric chloride, chromic oxide, Rose Bengal, 98%, ethanol 99.7%; toluene 99.8%; were supplied from Sigma/Aldrich and Chem Supply (Australia).

### 2.2. Synthesis of Nanoporous Alumina Membrane (PA)

PA membranes were prepared using a two-step anodization process as previously described [19-20]. Briefly, the Al foil was cleaned in acetone and then electrochemically polished in a 1:4 volume mixture of perchloric acid and ethyl alcohol by a constant voltage of 20 V for 2 minutes to achieve a mirror finished surface. Anodization process was performed using an electrochemical cell equipped with a cooling stage at a temperature of −1 °C. The first anodization step was performed under 40–80 V for 2–8 h in 0.3 M oxalic acid. Afterwards, the formed porous oxide film was chemically removed by a mixture of 6 wt % of phosphoric acid and 1.8% chromic acid for a minimum of 3–6 h at 75 °C. The second anodization step was performed under the same condition as the first anodization. The thickness of prepared porous layer is controlled by anodization time (2 h to 8 h) and the pore diameters by anodization voltage (from 40 V to 100 V). After anodization, the remaining Al layer was removed from the oxide films using cupric chloride/hydrochloric acid solution followed by chemical etching in 5% phosphoric acid at 25 °C for 2–3 h to remove the bottom barrier layer of PA (pore opening), and prepare free-standing PA membrane with through hole morphology. The etching process was controlled by time, but the process was also additionally monitored with the method for controlled dissolution of the bottom barrier layer using a two-electrode system as described previously [19-20].

### 2.3. Synthesis of CNT/PA Composite Membrane

The synthesis of CNT inside of pores of PA membranes was performed in custom designed chemical vapor deposition (CVD) system which consist of tubular electric furnace equipped a quartz tube (42.75 mm in diameter, 1,000 mm long), temperature controller, gas controller and particle generator [21]. Carbon precursors were introduced into the reaction tube as ultra fine particle aerosols by utilizing a particle generator with argon as carrier gas (flow rate 1 L/min^−1^). In this work different experimental conditions including carbon precursors (with catalyst and catalyst-free), reaction temperature and reaction time were explored to optimize fabrication process and the quality of fabricated CNT/PA membranes. Ferrocene/toluene (catalyst based) and, toluene/ethanol, toluene, ethanol, phenothiazine, pyridine (catalyst-free) were explored as carbon precursors. The influence of reaction temperature from 600 °C to 950 °C was for selected precursors. The 850 °C was used as optimal temperature for the most experiments presented in this work. The reaction time for CNT growth was varied from 20 minutes to 2 h. When growth process was completed the tube furnace was slowly cooled to room temperature (cooling time was approximately 24 h).

### 2.4. Characterization of Prepared Membranes

The structural characterization of prepared PA and CNT/PA membranes was performed by scanning electron microscopy(SEM) using XL30ESEM from Philips and transmission electron microscopy (TEM) by utilising a Philips CM 200 TEM. Before inserting sample grids into the microscope, the samples were sonicated in ethanol, then dropped in the cupper grids and dried at room temperature.

X-ray defraction (XRD) patterns were acquired using a PanAlytical X' Pert Pro diffractometer with Co Kα source. CoKa radiation of 1.79026 Angstroms was applied.

The determination of molecular transport through fabricated CNT/PA membranes was performed using a U-tube permeation cell in which the membrane separates two half-cells; the feed cell and the permeate cell [22]. The hydrophilic dye, Rose Bengal (RB), was used as the model molecule to probe the transport properties of the CNT/PA membranes. The diffusion of the dye from the feed cell to the permeate cell was continuously monitored with a UV-Vis fibre optic spectrophotometer (Ocean Optics, USA) at 552.20 nm. Molecular flux of the dye through the membranes was determined.

## 3. Results and Discussion

### 3.1. Fabrication and Characterisation of PA Membranes Used as Template

**Figure 2:**
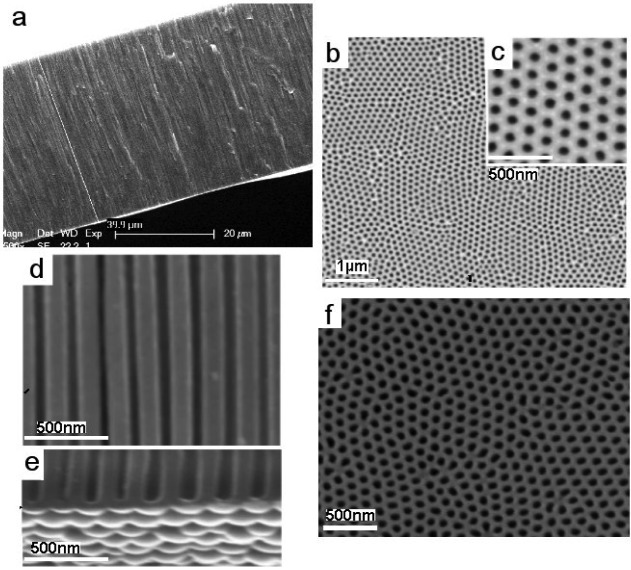
SEM images of PA membranes prepared by electrochemical anodisation of Al foil showing. (a) Cross-section of whole membrane structure; (b-c) top surface of pores; (d) high-resolution cross-sectional image of PA membranes showing straight channel of pore structures; (e) bottom surface with closed pores before pore opening; and (f) the bottom pore surface after pore opening.

The typical structures of PA membranes, prepared by two-step electrochemical anodization of aluminium in 0.3 M oxalic acid are presented in Figure 2. The cross-sectional SEM image of PA membrane shows a perfectly straight and densely packed array of nanopores across the whole structure (Figure 2a). The thickness of the PA membrane is about 40 μm (Figure 2a), but by selecting the anodization time (from 20 min to 8 h) we prepared PA membranes with desired thickness ranging from <5 μm to > 100 μm. SEM images of the top PA surface (Figure 2b-c) shows pore structures with diameters about 50 ± 10 nm and hexagonal arrangements of the pore cells. The pore diameters from 20 nm to 300 nm can be controlled by varying the anodization potential (20 V to 300 V) and selecting different electrolyte (sulphuric acid, oxalic acid and phosphoric acid) but in this work results on PA membrane prepared by anodization in oxalic acid are presented. Figure 2d presents a high-resolution SEM image of pore structure showing pore with perfectly flat channels. The bottom surface of self-supporting PA membranes before removal of the barrier layer on the bottom is presented in Figure 2e. The bottom surface of the PA membranes after removing of underlaying Al and removal of barrier oxide layer is presented in Figure 2f. In this work we mainly used PA with through-hole morphology using pore opening process but also PA with closed pores were used to demonstrate that the growth of CNT is possible on PA with closed pores.

### 3.2. Fabrication and Characterisation of CNT/PA Membranes Using CVD Process with Catalyst and Catalyst-Free Carbon Precursor

**Figure 3:**
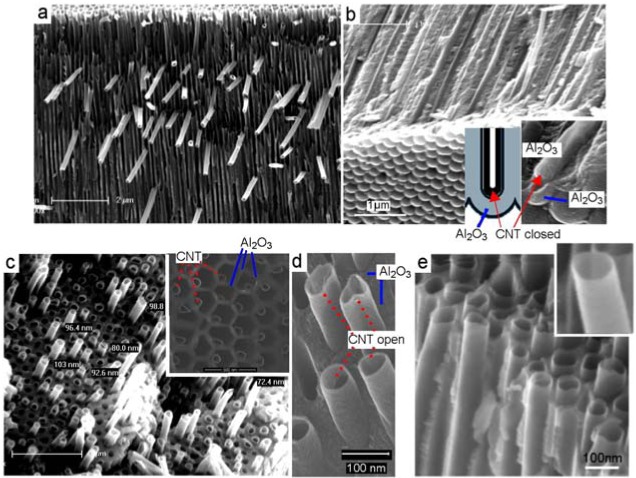
SEM images of carbon nanotube (CNT) synthetised inside of pores of PA membrane. (a) cross-sectional image of prepared CNT/PA composite membrane showing CNT inside of pores; (b) a cross-sectional image from the bottom in case when PA bottom was closed. Inset shows scheme of closed pores; (c) CNT structure inside of pores obtained from fractured membranes. Inset show CNT after partial removal of oxide; (d-e) high resolution images of CNT inside of pore showing that CNTs have identical shape as pore structures.

In a typical experiment, a carbon precursor ferrocene/tolune (catalyst based) or toluene/ethanol (catalyst-free) was transported by a carrier gas and decomposed within hot tube furnace (850 °C) where PA membrane is inserted. SEM images in Figure 3 show structure of CNT/PA composite membranes prepared by CVD growth of CNT inside of pores of PA membrane using catalyst based carbon precursor (ferrocene/toluene). Cross-sectional image of whole membrane (Figure 3a-b) clearly shows the presence of CNT inside of pores across all PA structure. The bottom part of CNT/PA (Figure 3b) shows the formation of CNT with closed CNT in case when PA with closed pores (Figure 2c-e) was used. When PA with through-pore morphology is used CNT/PA membrane with opened bottom is formed. Both diameter and the shape of the CNTs formed inside the PA template pores during CVD is the same as the pore structure of PA template (Figure 3c-e). Hence, a general feature of PA, its versatility to control the diameter of the CNTs, becomes apparent. Using PA membranes with different pore diameters fabricated under different anodization condition we demonstrated the preparation of CNT/PA membranes with range of internal diameters from <10 nm to >200 nm.

**Figure 4:**
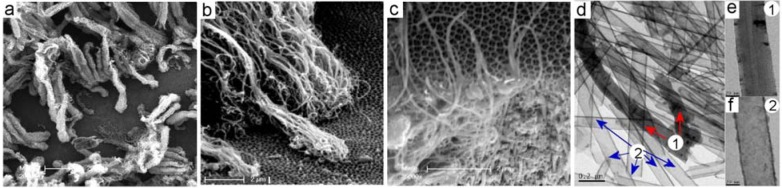
(a-b) Overgrowth of CNT on the top of PA membranes caused by catalytic reaction on the PA surface; (c) overgrowth of CNT caused by ferrocene catalytic reaction from the pores; (d) TEM image of CNT prepared after liberation of nanotubes from template showing two different types of CNT (1) iron catalyst induced growth on surface and (2) CNT grown inside of pore; (e) high resolution TEM image of catalyst growth CNT; and (f) pore growth CNT.

Although robust and well defined CNT/PA membranes with compact CNT structure were formed inside of PA pores we observed unwanted overgrowth of CNT on the top of PA surface in case when catalyst-based carbon precursor was used. Two different overgrowth processes of CNT were detected during this process. One process is where CNT started to grow by catalytic process from the surface of PA membranes (Figure 4a-b). In this case very dense CNT brushes with length >10 μm were sporadically formed across PA surface. In the second process the overgrowth of CNT was observed from CNT grown inside of pores. The overgrowth of single and isolated CNTs was observed on the PA surface as result that the CNT growth process is not terminated on the end of pores (Figure 4c). Interestingly this process was detected sporadically and on isolated locations at PA surface. Why this process is occurred and why CNT didn't stop to grow on the end of pores is not clear at this stage. SEM and TEM characterisation of CNT formed on the surface and inside of pores is performed to see difference of their structures. TEM image in Figure 4d show both CNT structures formed by catalytic process on the PA surface marked as (1) and CNT formed inside of pores marked as (2). Results obviously show differences between these structures that confirm different mechanism of their growth. CNT grown on the PA surface (Figure 4e) shows significantly thicker walls (∼15 nm) and better crystalinity of graphitic layers. The layered structures of CNT prove that formed CNTs are MWNT. The morphology of these structures is very similar to MWCNT growth by Fe nanoparticles from planar surface reported in previous studies [23]. Contrarily, the CNTs formed inside of pores show different morphology with thinner walls (4–5 nm) and poorer crystalinity. The multi graphitic structure of CNT wall is not clearly seen but it is likely that CNT are multilayerd. This result can be explained by ferrocene decomposition reaction (five-step reaction mechanism) and the formation of aromatics is described by irreversible kinetics without any agglomeration.

**Figure 5:**
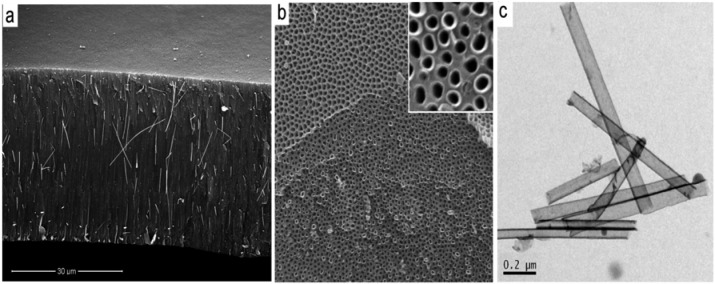
CNT/PA membranes prepared by catalyst free carbon precursor (toluene/ethanol). (a) cross-sectional SEM image of whole membrane; (b) Image from the top surface showing that the growth of the CNT is terminated on the top without of overgrowth; and (c) TEM image of liberated CNT grown inside of pores.

The uncontrolled growth of CNT outside of pores is a main disadvantage of this method which involves catalyst based carbon precursors. Therefore to avoid this problem and prepare CNT/PA with clean top surface we explore to use catalyst free carbon precursors. This idea is based on previous studies which shows that alumina surface of PA can act as catalyst and catalyst is not necessary to be included in carbon precursor [24]. Several carbon precursors were explored including ethanol, toluene, toluene/ethanol and pyridine. SEM characterisation results confirm preparation of CNT/PA composite membranes using these percussors, which confirms that alumina surface act as catalyst, and catalyst is not necessary used with carbon precursor. Figure 5 presents a typical SEM image of CNT/PA membrane showing a cross-sectional membrane structure and the top surface. The top surface is clean over all membrane surfaces which prove that overgrowth of CNT is eliminated using catalyst-free carbon precursor.

We observed some differences in the thickness of the wall of prepared CNT using different carbon precursor (e.g., ethanol *vs* toluene) and the best results obtained using toluene/ethanol. TEM image presents CNT structure grown inside of pores showing the wall thickness of 5–7 nm and not well defined graphitic layers similarly as seen on CNT grown with catalyst based precursor (ferrocene/toluene). Influence of temperature from 600–950 °C was investigated and the best results using CVD process in temperature range 800–850 °C.

**Figure 6:**
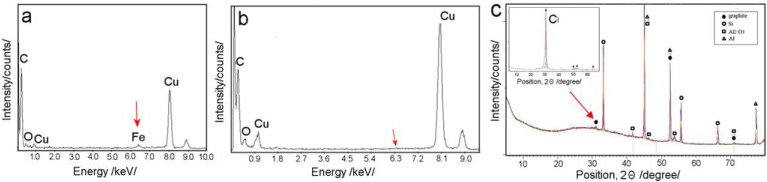
EDX analysis of CNT synthesised in PA with catalyst (ferrocene/toluene) carbon precursor taken from (a) the top of the PA surface and (b) the inside of PA pores and (c) XRD spectrum CNT inside of PA that indicates a small carbon peak (arrow). Inset shows XRD spectrum of CNT liberated from PA template that shows very strong carbon peak (ferrocene/toluene).

EDX characterization of CNT liberated from PA template was performed in order to get more information about their chemical composition. EDX graph (Figure 6a) obtained from CNT grown on PA surface using ferrecene/toluene precursors show the presence of Fe peak which verify that this process is catalysed by iron from ferrocene. It was surprising that Fe peak is not seen on CNT formed inside of PA pores (Figure 6b). This results lead to conclusion that catalysis by alumina surface is more important that catalysis by iron from ferrocene and to use catalyst is not necessary. This conclusion is supported by EDX results from CNT obtained by catalyst-free precursors (ethanol, toluene, toluene/ethanol) which show the formation of CNT without of presence of catalysts.

XRD studies of prepared CNT/PA were performed in order to get more information about their crystal structures. Figure 6c shows XRD spectrum from as-produced CNT/PA membrane *(ferrocene/toluene)*(4.5 kW Co Kα, λ = 1.789 Å). The graphs shows the almost complete absence of the (002) peak and a broadened peak combined with an extremely low intensity at 30. This result is known to be an indication that CNTs are straight and vertically arrayed inside the AAO template, hence the (002) faces (along the tube axis) of each nanotube are also perpendicular to the top array of nanotubes [23,25]. XRD graph shows (100) peak at 52.5° (2θ) and the (110) peak at 71.5°, is related to a crystal spacing of 0.35 and 0.153 nm respectively. No peaks were detected to the presence of any Fe_2_O_3_ or FeC_3_ which again confirm results from EDX and TEM showing CNT formed inside of PA pores are catalyst free. The benefit of utilising PA template for synthesis of CNTs is obvious in preparing CNT with higher level of purity which is important for many other applications.

**Figure 7:**
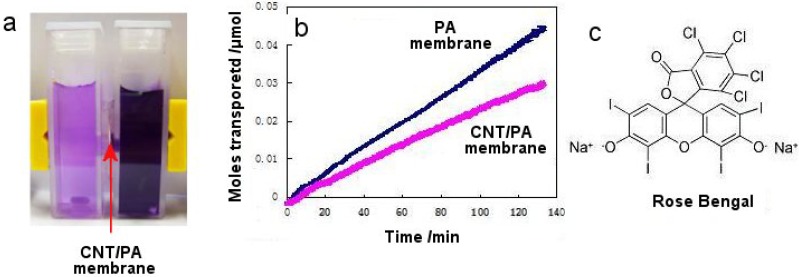
a) Experimental set-up for measurement of transport properties of prepared CNT/PA membranes; b) flux of model molecules (Rose Bengal) through membrane determined before (PA) and after CNT synthesis (CNT/PA); and c) formula of Rose Bengal.

Finally, the transport properties of prepared CNT/PA membranes were explored using Rose Bengal (RB) dye as model of molecule with hydrophilic properties. Figure 7 represents the transport data of RB dye through PA membrane before and after CNT formation (CNT/PA membrane). These data confirms that the hydrophilic RB dye is allowed to pass freely though CNT membranes but with lower flux than with uncoated PA membrane. This result is not surprising because diameter of these CNT membrane was very large (<40 nm) in comparison to size of dye molecule (1.5 nm). To achieve enhanced transport properties reported by previous studies [11-13] related to unique low-friction properties and hydrophobic properties of CNT surface it is crucial to decrease the size of CNT diameter relevant to the size of transported molecules. This study is underway using the CNT/PA membranes with smaller CNT diameters and series of model molecules with different size and different interfacial properties.

## 4. Conclusions

The fabrication of CNT/PA membranes using CVD process with catalyst based and catalyst-free carbon precursors was demonstrated. The CNT/PA membranes with desired CNT morphology and diameters from <20 nm to >200 nm were prepared by selecting PA membranes with preferred pore dimensions. The problem with overgrowth of CNT using catalytic decompositions toluene/ferrocene was eliminated by the use catalysis-free precursors such as toluene/ethanol showing that alumina surface of PA membrane can act as catalyst and generate the grown of CNT inside of pores. TEM, EDX and XRD results confirmed that in both cases CNT formed inside of pores are extremely pure and catalyst-free, which is not possible to achieve using other synthetic methods. The results from transport properties of prepared CNT/PA membranes suggested that the size of CNT should be decreased to be comparable with the size of transported molecules.
